# KRAS Footprints in the Skin: Leveraging Targeted Therapy for Unresectable Intra‐Cerebral AVM

**DOI:** 10.1111/pde.70126

**Published:** 2025-12-29

**Authors:** Donglin Zhang, Jennifer M. Kwon, Beverly Aagaard‐Kienitz, Susan L. Rebsamen, Sarah E. Mc Dermott, Lisa M. Arkin

**Affiliations:** ^1^ Department of Dermatology University of Wisconsin School of Medicine Madison Wisconsin USA; ^2^ Department of Neurology University of Wisconsin School of Medicine Madison Wisconsin USA; ^3^ Department of Neurological Surgery University of Wisconsin School of Medicine Madison Wisconsin USA; ^4^ Department of Radiology University of Wisconsin School of Medicine Madison Wisconsin USA; ^5^ Division of Hematology, Oncology, Transplant and Cellular Therapy University of Wisconsin School of Medicine Madison Wisconsin USA

**Keywords:** arteriovenous malformations, central nervous system vascular malformations, intracranial arteriovenous malformations, KRAS protein, vascular malformations

## Abstract

A 15‐year‐old female with a longstanding, unresectable intracerebral arteriovenous malformation (AVM) involving the bilateral thalami and basal ganglia presented with progressive neurologic decline. Given the inaccessibility of the intracranial lesion, a lipomatous scalp nodule overlying the AVM was biopsied for molecular testing and revealed a somatic mosaic *KRAS* p.G12D variant, the most common variant detected in sporadic brain AVMs. Targeted therapy with the MEK inhibitor trametinib was initiated, but the treatment course was complicated by cutaneous toxicity and ongoing neurologic deterioration. This case illustrates that extracranial tissue in the skin can serve as a surrogate for molecular diagnosis in unresectable brain AVMs, underscoring the diagnostic and therapeutic importance of dermatologic assessment in complex vascular anomalies.

To the Editors,

1

A 15‐year‐old female presented to the multidisciplinary vascular anomalies clinic with a cerebral arteriovenous malformation (AVM) involving the bilateral thalami and basal ganglia, initially discovered 8 years prior during evaluation for meningitis. Her course was notable for progressive neurologic decline, including worsening fatigue, gait instability, urinary incontinence, and right‐sided dystonia. Magnetic resonance angiography (MRA) confirmed an extensive, enlarging AVM with ventriculomegaly (Figure 1). Dermatologic examination revealed a large, lipomatous nodule on the midline vertex scalp (Figure 2), located directly over the AVM on MRI. No cutaneous vascular malformations were present, and the family history was negative for AVMs or vascular anomalies.

**FIGURE 1:**
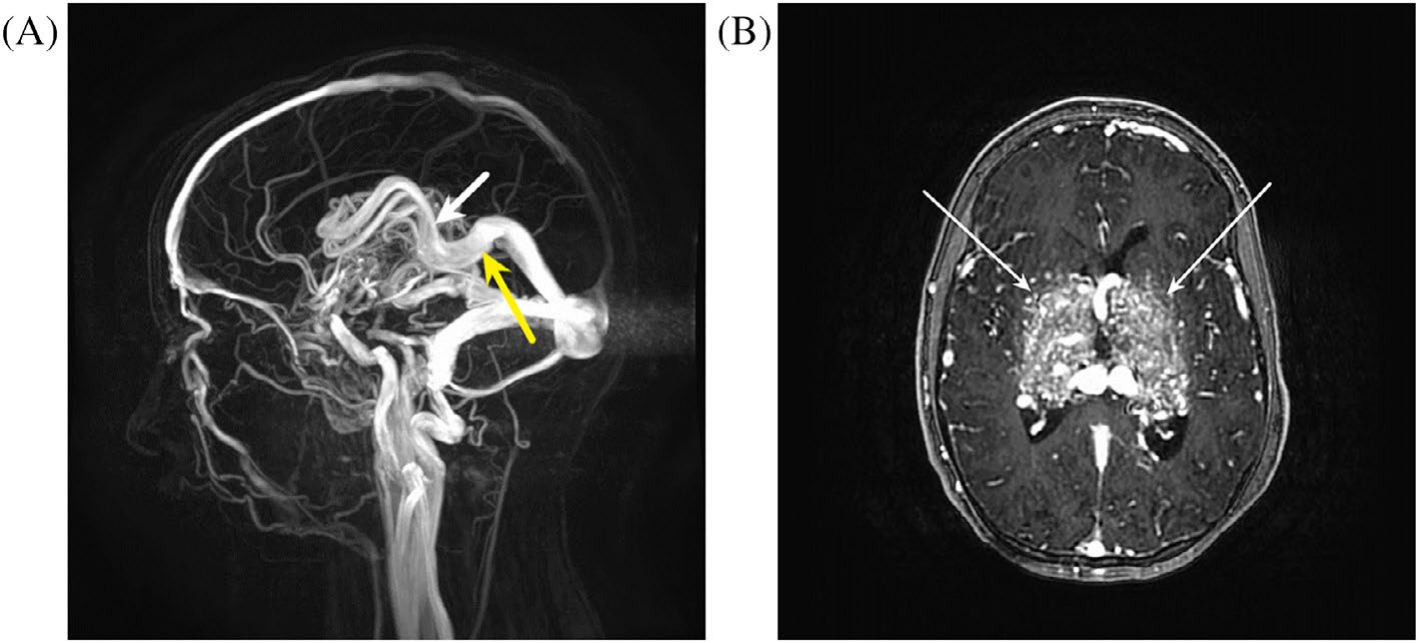
(A) Volumetric 3DPCA MRA post‐contrast. MRA demonstrates a bithalamic arteriovenous malformation with arterial feeders from both anterior and posterior circulation, with a dominant deep venous drainage pattern including the internal cerebral veins (white arrow) and vein of Galen (yellow arrow). (B) Volumetric T1 Stealth Bravo post‐contrast. Imaging demonstrates a complex arteriovenous malformation involving the bilateral thalami (arrows).

**FIGURE 2:**
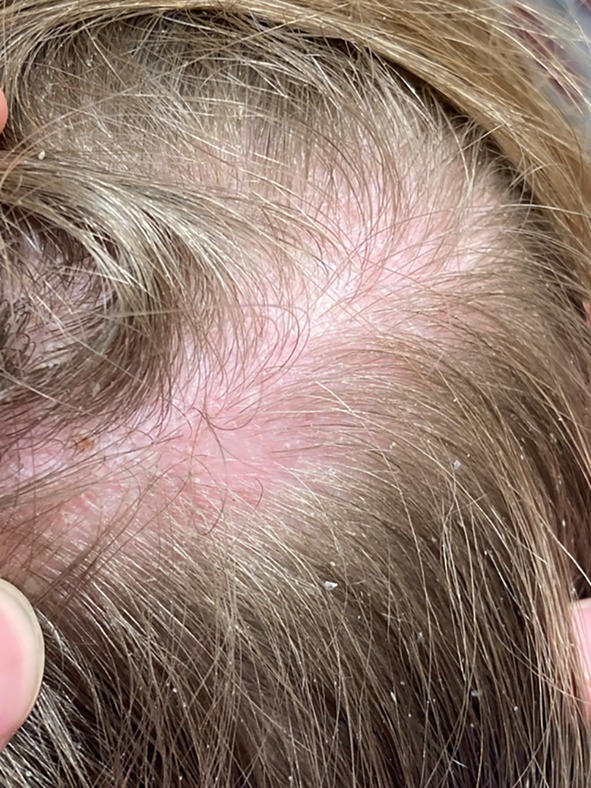
Subcutaneous soft mass measuring ~1.5 cm by 5 cm on the posterior vertex scalp.

Due to the lesion's deep location, surgical or radiologic intervention was contraindicated, and biopsy of the AVM itself posed unacceptable risk. The scalp nodule—suspected to share the AVM's genetic driver—was biopsied for molecular analysis. Next‐generation sequencing revealed a *KRAS* p.G12D variant (13.4% allele frequency) [1]. Given the presence of a targetable *KRAS* mutation and reports of efficacy in extracranial AVMs, oral trametinib (a MEK inhibitor) was initiated and titrated to 1.5 mg daily. Treatment was complicated by acneiform eruption, cheilitis, and chronic paronychia, limiting further dosage increases. Despite therapy, neurologic decline continued with worsening fatigue, dystonia, and dysarthria. After 13 months, repeat MRI/MRA showed stable AVM size and configuration, but perfusion studies demonstrated reduced global perfusion, consistent with her clinical deterioration. Trametinib was discontinued due to toxicity and lack of improvement. Subsequent thalidomide therapy was ineffective, and the patient succumbed to her disease 6 months later.

Somatic *KRAS* mutations, most commonly at codon 12 (e.g., *p.G12D, p.G12V*), are found in ~60% of sporadic brain AVMs [1]. These activating variants drive endothelial proliferation and abnormal vessel formation through downstream RAF–MEK–ERK and PI3K–AKT–mTOR signaling. In animal models, endothelial expression of *KRAS p.G12D* or *KRAS p.G12V* induces AVM formation, with enhanced ERK1/2 signaling and angiogenic gene expression [1, 2]. These effects are partially reversible with MEK inhibition, supporting a rationale for targeted therapy. Clinical experience with MEK inhibitors in brain AVMs is limited to isolated case reports [3, 4].

Our patient's AVM was not amenable to procedural intervention while her progressive symptomatic decline presented an urgent need for medical treatment. The same *KRAS* p.G12D variant detected in brain AVMs was present in an overlying lipomatous scalp lesion—a finding not previously reported. While KRAS variants have been described in lipomas associated with encephalocraniocutaneous lipomatosis [2], this case uniquely links a lipoma and a brain AVM via shared somatic mutation. In this case, MEK inhibition led to radiographic disease stabilization of her AVM, but worsening perfusion studies aligned with her continued neurologic decline. It is unclear whether trametinib contributed to some disease stabilization during the treatment period, or whether earlier initiation of therapy at diagnosis could have altered the outcome, underscoring the need for further study of targeted approaches. This report highlights the value of meticulous dermatologic examination and molecular testing in patients with unresectable AVMs, as accessible cutaneous tissue may harbor the same pathogenic variant and facilitate precision‐based approaches to therapy.

## Consent

Patient consent obtained and is on file for the publication of all patient photographs and medical information to be published in print and online, with the understanding that this information may be publicly available.

## Conflicts of Interest

The authors declare no conflicts of interest.

## Data Availability

Data sharing not applicable to this article as no datasets were generated or analyzed during the current study.
